# Carbapenem-resistant gram-negative bacterial infections and risk factors for acquisition in a Kenyan intensive care unit

**DOI:** 10.1186/s12879-024-09256-6

**Published:** 2024-05-23

**Authors:** Jane Wairimu Maina, Jeniffer Munyiva Mutua, Abednego Moki Musyoki

**Affiliations:** 1 Department of Medical Laboratory Sciences, Kenyatta University, Nairobi, P.O BOX 43844-00100, Kenya; 2 Department of Medical Laboratory, The Nairobi West Hospital, Nairobi, P.O BOX 43375-00100, Kenya; 3 Department of Laboratory Medicine, Kenyatta National Hospital, Nairobi, P.O Box 20723-00202, Kenya

**Keywords:** Carbapenem-resistant Gram-negative bacteria, Multiple antibiotic resistance index, Multidrug resistance, Risk factors

## Abstract

**Background:**

Carbapenem-resistant Gram-negative bacteria (CR-GNB) are a critical public health threat globally; however, there are inadequate surveillance data, especially in intensive care units (ICU), to inform infection prevention and control in many resource-constrained settings. Here, we assessed the prevalence of CR-GNB infections and risk factors for acquisition in a Kenyan ICU.

**Methods:**

A hospital-based cross-sectional study design was adopted, recruiting 162 patients clinically presenting with bacterial infection after 48 h of ICU admission, from January to October 2022 at the Nairobi West Hospital, Kenya. Demographics and clinical data were collected by case report form. The type of sample collected, including blood, tracheal aspirate, ascitic tap, urine, stool, and sputum depended on the patient’s clinical presentation and were transported to the hospital Microbiology laboratory in a cool box for processing within 2 h. The samples were analyzed by cultured and BD Phoenix system used for isolates’ identity and antimicrobial susceptibility.

**Results:**

CR-GNB infections prevalence was 25.9% (42/162), with *Klebsiella pneumoniae* (35.7%, 15/42) and *Pseudomonas aeruginosa* (26.2%, 11/42) predominating. All isolates were multidrug-resistant (MDR). *P. aeruginosa* and *A. baumannii* were 100% colistin-resistant, while *K. pneumoniae* (33.3%) was tigecycline-resistant. History of antibiotics (aOR = 3.40, *p* = 0.005) and nasogastric tube (NGT) use (aOR = 5.84, p = < 0.001) were the risk factors for infection.

**Conclusion:**

Our study highlights high MDR- and CR-GNB infections in ICU, with prior antibiotic exposure and NGT use as risk factors, and diminishing clinical value of colistin and tigecycline. In this study setting and beyond, strict implementation of antimicrobial stewardship programs and adherence to infection prevention and control through monitoring, evaluation and feedback are warranted to curb CR-GNB infections, especially among the risk groups.

## Background

Carbapenem-resistant Gram-negative bacteria (CR-GNB) presents significant challenges in clinical practice [1]. These organisms are World Health Organization (WHO) critical priority pathogens, depending on the urgency with which new antibiotics are needed [2]. CR-GNB pathogens are often multidrug-resistant (MDR) with limited treatment options, increased morbidity, healthcare cost and mortality [2]. Carbapenems are considered antibiotics of ‘the last resort’ for infections caused by MDR-GNB. There are several mechanisms of carbapenem resistance, mainly limiting the uptake of a drug, modification of a drug target, inactivation of a drug, and active efflux of a drug [3].

Colistin and tigecycline are the first-line treatment options for infections caused by carbapenem-resistant (CR) bacteria, particularly in many resource-constrained countries with limited access to newer therapeutic options; however, uncertainties on their efficacy still exist due to emerging resistance, even when combined with other antimicrobials. The newer treatment options for CR bacterial infections, such as ceftazidime-avibactam, ceftolozane-tazobactam, meropenem-vaborbactam, imipenem-cilastatin-sulbactam, plazomicin, eravacycline, and cefiderocol, are faced with challenges of insufficient high-quality clinical data, delayed susceptibility testing methods approval, antibacterial spectra complexity, and acquisition costs [4].

Intensive care units (ICUs) are recognized as hotbeds for MDR pathogens acquisition and spread because the ICU-admitted patients are usually severely ill and have frequent invasive procedures, including intubation, mechanical ventilation, and vascular access. Frequently, ICU-admitted patients have reduced immunity following trauma, surgery, and sepsis and also due to impaired protective mechanisms that include cough and swallowing reflexes, gastric acid secretion, and normal flora [5]. The burden of ICU-acquired infections is higher in lower-middle-income countries (LMICs) compared to high-income countries [6]. Therefore, continuous and systematic-evidence-based surveillance, in line with the global action plan on AMR [7], is warranted in ICUs to inform infection prevention and control measures and optimized use of antimicrobials.

Clinical laboratories are crucial in generating antibiograms for evidence-based antimicrobial selection by clinicians and AMR surveillance. However, these facilities in many resource-limited countries are inadequate and lack the capacity for microbiology, with the majority of patients likely to receive inappropriate antibiotic prescriptions. A recent study in Kenya shows that only 0.1% of 1505 patients in 14 public hospitals were treated based on antibiograms, and 46.4% inappropriately received antibiotics [8]. This clinical practice has critical implications for the emergence and spread of MDR organisms. MDR pathogens, including CR strains, are a growing health problem in Kenya [9, 10]; however, data on CR-GNB, especially in our ICUs, is limited. Here, we determined the prevalence CR-GNB infections and risk factors for acquisition among patients admitted to the ICU.

## Methods

### Study area, design, and population

We conducted this cross-sectional study between January and October 2022 among consecutive patients admitted to the intensive care unit (ICU) at the Nairobi West Hospital (NWH), Kenya. The NWH is a 400-bed capacity (including 18 ICU beds) private tertiary hospital that receives patient referrals from all over the country. The study included patients clinically presenting with a bacterial infection (persistent fever, swollen lymph nodes, chills and sweats, confusion, cloudy and smelly urine, increased heart rate, difficulty breathing, persistent cough, vomiting, diarrhoea, wound that is red, hot, swollen, or has pus) and excluded those admitted for less than 48 h. The treating physician’s clinical judgement guided the type of sample collected from each patient. The authors sought informed written consent from each participant through a close relative or a legally appointed family representative and followed the Declaration of Helsinki, observing the well-being of patients and prompt sharing of results with the treating clinicians. The Kenyatta University Ethical Review Committee granted the study ethical clearance (Protocol no. PKU/2395/11,531).

### Data and clinical samples collection

The participants’ demographic and clinical presentation data were collected using a case report form. The clinical sample types depended on the patient’s clinical presentation and were collected following standard bacteriological procedures [11], as previously described by Maina and others [12]. A qualified nurse collected the tracheal aspirate and ascitic tap samples into sterile containers. Swab samples were collected using sterile swabs (Delta lab, Spain), whereas urine samples were collected aseptically from a catheter collection port using a needle into 20 mL sterile screw-capped universal containers (Delta lab, Spain). Stool and sputum samples were collected into a sterile polypots (Delta lab, Spain). For blood samples, we obtained 8–10 mL of participants’ blood using a needle and syringe into BD BACTEC™ Blood Culture Media (BD Diagnostics, Sparks, MD, USA). All samples were uniquely labeled, blood cultures held at room temperature and other samples in a cool box and transported to the NWH Microbiology laboratory for processing within 2 h [12].

### Isolation, identification and antimicrobial susceptibility testing

Bacterial isolation followed the standard operating procedures in bacteriology [13]. We inoculated stool samples on MacConkey agar and Xylose Lysine Deoxycholate agar (XLD agar), while pus swabs, sputum, catheter tips, and tracheal aspirates on MacConkey agar, sheep blood agar, and chocolate blood agar (all culture media from Hi Media Laboratories LLC, India); and incubated overnight at 37 °C under both ambient air and 5% CO2 conditions. Urine samples were inoculated onto cysteine–lactose electrolyte-deficient agar (CLED) (HI Media Laboratories LLC, India) and incubated overnight at 37 °C. Blood samples were cultured in the BD BACTEC™ Automated Blood Culture System (BD Diagnostics, Sparks, MD, USA) at 36 °C for up to 5 days, and positive-flagged samples were sub-cultured on MacConkey agar, sheep blood agar, and chocolate agar, (Hi Media Laboratories LLC, India), then incubated overnight at 37 °C under both ambient air and 5% CO2 conditions [12].

The isolates’ identification and antimicrobial susceptibility testing were performed using the BD Phoenix system (BD Diagnostics, Sparks, MD, USA) and following the manufacturer’s instructions. Antibiotics selection and interpretation of the isolates susceptibility followed the CLSI guidelines [13]. The tested antibiotics were amoxicillin/clavulanic acid (4/2–16/2 µg/ml), ampicillin (4–16 µg/ml), piperacillin/tazobactam (4/4–64/4 µg/ml), trimethoprim/sulfamethoxazole (1/19–4/76 µg/ml), nitrofurantoin (16–64 µg/ml), gentamicin (2–8 µg/ml), amikacin (8–32 µg/ml), ceftriaxone (1–32 µg/ml), cefazolin (4–16 µg/ml), cefotaxime (4–16 µg/ml), ceftolozane/tazobactam (1/4–8/4 µg/ml), ceftazidime (2–16 µg/ml), cefepime (1–16 µg/ml), tigecycline (1–4 µg/ml), ciprofloxacin (0.5–2 µg/ml), levofloxacin (1–4 µg/ml), meropenem (0.5–4 µg/ml), ertapenem (0.25–2 µg/ml), imipenem (0.25–4 µg/ml) and colistin (1–4 µg/ml). *Pseudomonas aeruginosa* (ATCC 27,853) and *Escherichia coli* (25,922) were used as the standard control organisms [12].

We defined carbapenem resistance as resistance to either ertapenem (≥ 2 µg/ml), imipenem (≥ 4 µg/ml), or meropenem (≥ 4 µg/ml), whereas resistance to either ceftriaxone (≥ 4 µg/ml) or ceftazidime (≥ 16 µg/ml) as third-generation cephalosporin resistance [12, 13]. Isolates resistant to three or more antibiotic classes were considered multidrug-resistant (MDR) [9]. Multiple antibiotic resistance indices (MARI) were calculated as a/b, where a = number of antibiotics isolate was resistant to, b = the total number of antibiotics tested [12, 14].

### Statistical analysis

This study analyzed the data using the Statistical Package for the Social Sciences (SPSS) version 17.0 for Windows (IBM SPSS Statistics, IBM Corporation, Armonk, NY, USA). We analyzed data for normality and presented in figures and tables, categorical data in frequencies and percentages, and continuous data in means, medians, and interquartile ranges. Authors used binomial logistic regression analysis to determine the association between CR-GNB infections and patients’ socio-demographic and clinical characteristics. Any association with *p*-value ≤ 0.2 were further analyzed by multinomial logistic regression, with the statistical significance level set at *p* < 0.05 (95% Confidence Interval (95% CI)) and statistically significant associations bolded in Table 4  [12].

**Table 1:** MDR phenotypes among CR-GNB isolates

Isolate	Resistance patterns	No. of ABS classes	MARI	MDR phenotype n (%)
*Acinetobacter baumannii (5)*	GEN/ETP-IPM-MEM/CFZ-CXM-CAZ-CRO-FEP-C.T/AMP-AMC-TZP/SXT/NIT/CIP-LXV	7	0.85	1 (20)
	AMK- GEN/ETP-IPM-MEM/CFZ-CXM-CAZ-CRO-FEP-C.T/AMP-AMC-TZP/SXT/NIT/CIP-LXV	7	0.90	1 (20)
	AMK- GEN/ETP-IPM-MEM/CFZ-CXM-CAZ-CRO-FEP-C.T/AMP-AMC-TZP/ CST /SXT/NIT/CIP-LXV	8	0.95	2 (40)
	AMK- GEN/ETP-IPM-MEM/CFZ-CXM-CAZ-CRO-FEP-C.T/AMP-AMC-TZP/ CST /SXT/NIT	7	0.85	1 (20)
*Escherichia coli (8)*	ETP-IPM-MEM/CFZ-CXM-CAZ-CRO-FEP-C.T/AMP-AMC-TZP/ SXT/NIT/CIP-LXV	6	0.80	1 (12.5)
	AMK- GEN/ETP-IPM-MEM/CFZ-CXM-CAZ-CRO-FEP-C.T/AMP-AMC-TZP /SXT	5	0.75	1 (12.5)
	AMK/ETP-MEM/CFZ-CXM-CAZ-CRO-FEP-C.T/AMP-AMC-TZP/ CST /CIP-LXV	6	0.75	1 (12.5)
	ETP-MEM/CFZ-CXM-CAZ-CRO-FEP-C.T/AMP /SXT/NIT/CIP-LXV	6	0.65	1 (12.5)
	ETP-IPM-MEM/CFZ-CXM-CAZ-CRO-FEP-C.T/AMP/ CST /SXT/CIP-LXV	6	0.70	1 (12.5)
	GEN/ETP-MEM/CFZ-CXM-CAZ-CRO-FEP-C.T/AMP-AMC/SXT/CIP-LXV	6	0.70	1 (12.5)
	GEN/ETP-IMP-MEM/CFZ-CXM-CAZ-CRO-FEP-C.T/AMP-AMC/CST/SXT/CIP-LXV	7	0.80	1 (12.5)
	ETP-MEM/CFZ-CXM-CAZ-CRO-FEP-C.T/AMP/SXT/NIT/CIP	6	0.60	1 (12.5)
*Klebsiella pneumoniae (15)*	AMK- GEN/ETP-IPM-MEM/CFZ-CXM-CAZ-CRO-FEP-C.T/AMP-AMC-TZP/ CST /SXT/NIT/CIP-LXV/TIG	9	1.00	2 (13.3)
	ETP-IPM-MEM/CFZ-CXM-CAZ-CRO-FEP-C.T/AMP-AMC-TZP/ CST /SXT/NIT/CIP-LXV/TIG	8	0.90	1 (6.7)
	AMK- GEN/ETP-IPM-MEM/CFZ-CXM-CAZ-CRO-FEP-C.T/AMP-AMC-TZP/ CST /SXT/NIT/CIP-LXV	8	0.95	7 (46.7)
	AMK- GEN/ETP-IPM-MEM/CFZ-CXM-CAZ-CRO-FEP-C.T/AMP-AMC-TZP/SXT/NIT/CIP-LXV	7	0.90	1 (6.7)
	ETP-IPM-MEM/CFZ-CXM-CAZ-CRO-FEP-C.T/AMP-TZP/SXT/NIT/CIP-LXV/TIG	7	0.80	1 (6.7)
	GEN/ETP-IPM/CFZ-CXM-CAZ-CRO-FEP-C.T/AMP-AMC-TZP/ CST /SXT/NIT/CIP	8	0.80	1 (6.7)
	GEN/ETP-IPM/CFZ-CXM-CAZ-CRO-FEP-C.T/AMP/SXT/NIT/CIP-LXV/TIG	8	0.75	1 (6.7)
	ETP-IPM/CFZ-CXM-CAZ-CRO-FEP-C.T/AMP/ CST /SXT/CIP-LXV	6	0.65	1 (6.7)
*Pseudomonas aeruginosa (11)*	GEN/ETP-IPM-MEM/CFZ-CXM-CAZ-CRO-FEP-C.T/AMP-AMC-TZP/ CST /SXT/NIT/CIP-LXV/TIG	9	0.95	2 (18.2)
	AMK- GEN/ETP-IPM-MEM/CFZ-CXM-CAZ-CRO-FEP-C.T/AMP-AMC-TZP/ CST /SXT/NIT/CIP-LXV/TIG	9	1.00	3 (27.3)
	ETP-IPM-MEM/CFZ-CXM-CAZ-CRO-C.T/AMP-AMC/ CST /SXT/NIT/TIG	7	0.70	1 (9.1)
	GEN/ETP-IPM-MEM/CFZ-CXM-CAZ-CRO-FEP-C.T/AMP-AMC-TZP/ CST /SXT/NIT/CIP/TIG	9	0.90	1 (9.1)
	GEN/ETP-IPM/CFZ-CXM-CRO/AMP-AMC/ CST /SXT/NIT	7	0.55	1 (9.1)
	ETP-IPM-MEM/CFZ-CXM-CRO-FEP/AMP-AMC/ CST /SXT/NIT/TIG	7	0.65	1 (9.1)
	ETP-IPM-MEM/CFZ-CXM-CRO-/AMP-AMC/ CST /SXT/NIT/TIG	7	0.60	1 (9.1)
	GEN/ETP/CFZ-CXM-CRO-FEP/AMP-AMC/ CST /SXT/NIT/CIP-LXV/TIG	9	0.70	1 (9.1)

***ABS****antibiotics*, ***No***. *number*, ***MARI****multiple antibiotic resistance index*, ***MDR****multidrug-resistant*.

## Results

### Demographic and clinical characteristics of patients with GNB infections

The majority of the patients with Gram-negative bacteria (GNB) infections were: aged between 40 and 60 years old (46.7%), males (58.9%), not referred from other healthcare facilities (62.2%), and had: a history of antibiotic use (76.7%), invasive procedure (74.4%), and prior hospitalization history (62.2%) but not in ICU (98.9%), Table 2.

**Table 2:** Demographic and clinical characteristics of patients with GNB infections

Variable	Frequency (*N* = 90)	Percent (%)
***Age***		
< 18 years	7	7.8
19–39 years	31	33.4
40–60 years	42	46.7
> 60 years	10	11.1
***Gender***		
Male	53	58.9
Female	37	41.1
***Primary reason for admission***		
Respiratory tract infection	16	6.7
Cardiovascular disease	19	21.1
Cancer	11	12.2
Brain infection	4	4.4
Gastrointestinal infection	5	5.6
Kidney disorder	4	4.4
Fractures	7	7.8
Others	24	26.7
***Referral status***		
Referral	34	37.8
Non-referral	56	62.2
***Comorbidity***		
Yes	52	57.8
No	38	42.2
***History of antibiotic*** **use**		
Yes	69	76.7
No	21	23.3
***Invasive procedure*** **done**		
Yes	67	74.4
No	23	25.6
***Prior ICU admission***		
Yes	1	1.1
No	89	98.9
***Prior hospitalization***		
Yes	56	62.2
No	34	37.8
**NG tube**		
Yes	39	43.3
No	51	56.7

***NG- nasogastric***

### Distribution of CR-GNB in clinical samples

**Fig. 1:**
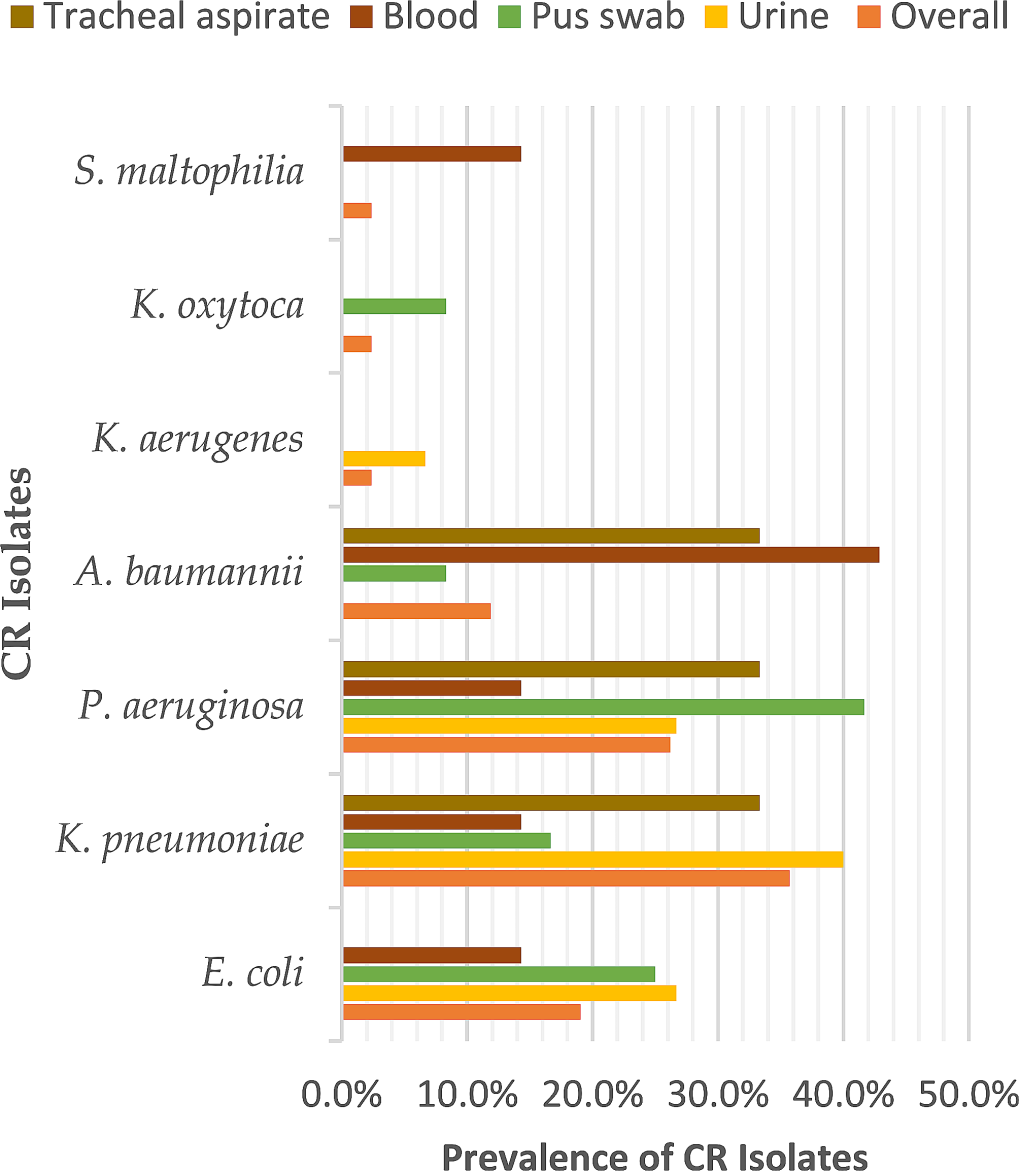
Distribution of CR-GNB. *% percentage*, ***No***. *number*, ***CR****carbapenem-resistant*, ***CS****carbapenem-sensitive*

In this study, 90 out of 162 (55.6%) patients had non-replicate Gram-negative bacterial infections. The prevalence of carbapenem-resistant Gram-negative bacteria (CR-GNB) was 46.7% (42/90), with *Klebsiella pneumoniae* (35.7%, 15/42) and *Pseudomonas aeruginosa* (26.2%, 11/42) as the most prevalent isolates overall, Fig. 1. In urine samples, *K. pneumoniae* predominated (6/15, 40%), while *Acinetobacter baumannii* (42.9%, 3/7) and *Pseudomonas aeruginosa* (41.7%, 5/12) were the most common pathogen in blood and pus swab samples, respectively. All *Acinetobacter baumannii* (5/5, 100%), 85% of *Pseudomonas aeruginosa* (11/13) and 54% of *K. pneumoniae* (15/28) were carbapenem-nonsusceptible, Fig. 1.

### Antimicrobial susceptibility profiles of CR-GNB

We observed third-generation cephalosporins resistance in all isolates of *Escherichia coli, Klebsiella pneumoniae, Pseudomonas aeruginosa*, and *Acinetobacter baumannii*, Table 3. Aminoglycosides resistance ranged from 25 to 100%, being highest among *A. baumannii isolates. Acinetobacter baumannii* and *P. aeruginosa* displayed 100% colistin resistance, Table 4. Additionally, *A. baumannii* showed 100% resistance to quinolones (LVX, CIP) tested but, together with *E. coli*, remained susceptible to tigecycline. Resistance to tigecycline was observed in *K. pneumoniae* (33.3%), *P. aeruginosa* (87.5%), Table 3. This study did not present the antimicrobial susceptibility profiles of *Stenotrophomonas maltophilia, Klebsiella oxytoca*, and *Klebsiella aerogenes* because they were only one isolate each.

**Table 3:** Antimicrobial susceptibility profiles of isolates

ABS class	ABS	P	E. coli (8)	K. pneumoniae (15)	P. aeruginosa (11)	A. baumannii (5)
Penicillin	AMP	R	100.0%	100.0%	100.0%	100.0%
		S	0.0%	0.0%	0.0%	0.0%
	AMC	R	62.5%	80.0%	100.0%	100.0%
		S	37.5%	20.0%	0.0%	0.0%
	PIP	R	37.5%	86.7%	50.0%	100.0%
		S	62.5%	13.3%	50.0%	0.0%
AMINO	AMK	R	25.0%	66.7%	75.0%	50.0%
		S	75.0%	33.3%	25.0%	50.0%
	GEN	R	37.5%	80.0%	69%	100.0%
		S	62.5%	20.0%	31%	0.0%
1GC	CFZ	R	100.0%	100.0%	100.0%	100.0%
		S	0.0%	0.0%	0.0%	0.0%
2GC/BLI	C/T	R	100.0%	100.0%	50.0%	100.0%
		S	0.0%	0.0%	50.0%	0.0%
3GC	CTX	R	100.0%	100.0%	100.0%	100.0%
		S	0.0%	0.0%	0.0%	0.0%
	CAZ	R	100.0%	100.0%	50.0%	100.0%
		S	0.0%	0.0%	50.0%	0.0%
	CRO	R	100.0%	100.0%	100.0%	100.0%
		S	0.0%	0.0%	0.0%	0.0%
4GC	FEP	R	100.0%	100.0%	75.0%	100.0%
		S	0.0%	0.0%	25.0%	0.0%
Sulfonamides	SXT	R	100.0%	100.0%	100.0%	100.0%
		S	0.0%	0.0%	0.0%	0.0%
Nitrofurans	NIT	R	37.5%	93.3%	100.0%	100.0%
		S	62.5%	6.7%	0.0%	0.0%
Quinolones	CIP	R	87.5%	100.0%	62.5%	100.0%
		S	12.5%	0.0%	37.5%	0.0%
	LVX	R	75.0%	93.3%	62.5%	100.0%
		S	25.0%	6.7%	37.5%	0.0%
Glycylcyclines	TGC	R	0.0%	33.3%	87.5%	0.0%
		S	100.0%	66.7%	12.5%	100.0%
Carbapenems	ETP	R	100.0%	100.0%	100.0%	100.0%
		S	0.0%	0.0%	0.0%	0.0%
	IMP	R	37.5%	93.3%	87.5%	100.0%
		S	62.5%	6.7%	12.5%	0.0%
	MEM	R	100.0%	93.3%	75.0%	100.0%
		S	0.0%	6.7%	25.0%	0.0%
Polymyxins	CST	R	37.5%	80.0%	100.0%	100.0%
		S	62.5%	20.0%	0.0%	0.0%

***AMP*** ampicillin, ***AMC*** amoxicillin-clavulanic acid, ***PIP*** piperacillin, ***AMK*** amikacin, ***GEN*** gentamicin, **CFZ-**cefazolin, ***C/T*** ceftolozane-tazobactam, ***CTX*** cefotaxime, ***CAZ*** ceftazidime, ***CRO*** ceftriaxone, ***FEP*** cefepime, ***SXT*** trimethoprime-sulfamethazole, ***NIT*** nitrofurantoin, ***CIP*** ciprofloxacin, ***LVX*** levofloxacin, ***TGC*** tigecycline, ***ETP*** ertapenem, ***IMP*** imipinem, ***MEM*** meropenem, ***CST*** colistin, ***S*** susceptible, ***R*** resistant, ***P****phenotype*, ***ABS****antibiotics*, ***AMINO****aminoglycosides*, ***1GC****first-generation cephalosporin*, ***2GC****second-generation cephalosporin*, ***3GC****third-generation cephalosporin*, ***4GC****fourth-generation cephalosporin*.

**Table 4:** Factors associated with CR-GNB infection

	GNB infection					
	CR n(%)	CS n(%)	cOR(95%CI)	*p*-value	aOR(95%CI)	*p*-value
**Age**						
< 18 years	2(4.8)	5(10.4)	*Ref*			
19–39 years	14(33.3)	17(35.4)	1.67(0.21–13.22)	0.629		
40–60 years	22(52.4)	20(41.7)	0.81(0.19–3.45)	0.775		
> 60 years	4(9.5)	6(12.5)	0.61(0.15–2.46)	0.484		
**Gender**						
Male	29(69.0)	24(50.0)	2.23(0.94–5.30)	0.087	1.92(0.71–5.18)	0.197
Female	13(31.0)	24(50.0)	*Ref*		*Ref*	
**Pathology**						
Respiratory tract infection	7(16.7)	9(18.8)	1.09(0.31–3.89)	0.897		
Cardiovascular disease	13(31.0)	6(12.5)	0.39(0.11–1.37)	0.143		
Cancer	3(7.1)	8(16.7)	2.26(0.48–10.64)	0.304		
Brain infection	2(4.8)	2(4.2)	0.85(0.10–7.04)	0.812		
Gastrointestinal infection	2(4.8)	3(6.3)	1.27(0.18–9.02)	0.877		
Kidney disorder	2(4.8)	2(4.2)	0.85(0.10–7.04)	0.812		
Fractures	2(4.8)	5(10.4)	2.12(0.34–13.13)	0.877		
Others	11(26.2)	13(27.1)	*Ref*			
**Referral status**						
Referral	17(40.5)	17(35.4)	1.24(0.53–2.91)	0.667		
Non-referral	25(59.5)	31(64.6)	*Ref*			
**Comorbidity**						
Yes	24(57.1)	28(58.3)	0.93(0.41–2.20)	0.539		
No	18(42.9)	20(41.7)	*Ref*			
**History of antibiotic use**						
Yes	37(88.1)	32(66.7)	3.70(1.22–11.23)	**0.024** ^*^	3.40(1.97–11.89)	**0.005** ^*^
No	5(11.9)	16(33.3)	*Ref*		*Ref*	
**Invasive procedure done**						
Yes	32(76.2)	35(72.9)	1.19(0.46–3.09)	0.811		
No	10(23.8)	13(27.1)	*Ref*			
**Prior ICU admission**						
Yes	1(2.4)	0				
No	41(97.6)	48(100)				
**Prior hospitalization**						
Yes	29(69.0)	27(56.3)	1.74(0.73–4.13)	0.277		
No	13(31.0)	21(43.8)	*Ref*			
**NG tube**						
Yes	28(66.7)	11(22.9)	6.73(2.66–17.05)	**< 0.001** ^**^	5.84(2.16–15.79)	**< 0.001** ^**^
No	14(33.3)	37(77.1)	*Ref*		*Ref*	

***ICU****Intensive care unit*, ***cOR****crudes Odds Ratio*, ***aOR****adjusted Odds Ratio*, ***CR****carbapenem-resistant*, ***CS****carbapenem-sensitive*, ***GNB***, *Gram-negative bacteria*, ***Ref****reference*, ***P****probability*, ***CI****confidence interval*, ***NGT****nasogastric tube*, * statistically significant at p < 0.05, **statistically significant at p < 0.001.

### MDR phenotypes

All the CR-GNB isolates were multidrug-resistant (MDR) and had multiple antibiotic resistance indexes ranging from 0.55 to 1.0, Table 4 .

### Factors associated with carriage of CR-GNB

Patients with a history of antibiotic use were three (3) times more likely to have CR-GNB isolate compared to those without the history (aOR = 3.40, 95% CI: 1.97–11.89, *p* = 0.005), while those using NG tube were about six (6) times more likely to be diagnosed with GNB infection (aOR = 5.84, 95% CI: 2.16–15.79, p = < 0.001), Table 4.

## Discussion

Carbapenem-resistant Gram-negative bacteria (CR-GNB) are an eminent global health challenge because of the limited treatment options and high mortality rates [1, 15]. The burden of CR-GNB is disproportionately higher in low and middle income countries (LMICs) compared to high-income countries [3]. Here in this study, the prevalence of CR-GNB was 46.7%, higher than previously reported in East Africa [16], the United States [17], and Central Asia and Europe [18]. These study findings suggest a higher burden of CR-GNB, in our study setting, possibly due to limited alternative therapeutic options, widespread and irrational use of carbapenem antibiotics, and the failure of existing treatments.

In the current study, carbapenem-resistant *Klebsiella pneumoniae* (CRKP, 35.7%, 15/42) and carbapenem-resistant *Pseudomonas aeruginosa* (CRPA, 26.2%, 11/42) were the most the prevalent isolates overall. Similarly, CRPA (4/42, 9.5%) and CRKP predominated in urine, while carbapenem-resistant *Acinetobacter baumannii* (CRAB, 42.9%, 3/7) was the most common pathogen in blood samples. In general, all *Acinetobacter baumannii* (5/5, 100%), 85% of *Pseudomonas aeruginosa* (11/13) and 54% of *K. pneumoniae* (15/28) were carbapenem-nonsusceptible. Our study findings corroborate with others among ICU-admitted patients in Egypt [19], South Korea [20]), Fuzhou, and China. *K. pneumoniae, P. aeruginosa* and *A. baumannii* are opportunistic pathogens known for their high frequency and diversity of antimicrobial resistance genes and are well adapted to hospital environments, where they frequently cause severe infections in hospitalized and immunodeficient persons.

The incidence of infections caused by third-generation cephalosporin-resistant (3GCR) and CR-GNB in ICU patients is rising [21]. We observed third-generation cephalosporins resistance (3GC-R) in all carbapenem-resistant *Escherichia coli* (CREc), CRKP, CRPA, and CRAB isolates, corroborating studies among ICU-admitted patients in Egypt [22, pp. 2011–2017]. 3GC-R was higher than reported in six German university hospitals [21]. The current study findings may suggest antibiotics overprescription, selecting resistant strains. Further, CRAB and CRPA, in our study, displayed 100% colistin resistance. Syed and others documented similar high colistin-resistance levels in ICUs at tertiary care hospitals in Karachi, Pakistan [23]. Colistin is considered ‘the drug of the last resort’ for many CR-GNB infections, with its frequent use in agriculture and pisciculture considered the cause of the rising resistance. In Kenya, 13% of farmers in Kiambu County used colistin in poultry feeds [24]. Colistin resistance is a growing problem, especially in developing countries, necessitating stringent infection control and comprehensive antimicrobial stewardship policies.

In the current study, *K. pneumoniae* (33.3%) and *P. aeruginosa* (87.5%) were resistant to tigecycline, but CRAB and CREc remained susceptible. *P. aeruginosa* is known to be intrinsically resistant to tigecycline, and in *E. coli* and *K. pneumoniae*, the resistance occurs due to AcrAB efflux pump overexpression [25]. The pharmacokinetic/pharmacodynamic properties of tigecycline and colistin suggest that these antimicrobial agents are among the most effective options in vitro in combating CR-GNB among critically ill patients with difficult-to-treat infections. The emergence of resistance to these antibiotics is, therefore, a significant issue that needs to be addressed, considering that the newly approved antimicrobial agents, such as ceftazidime/avibactam and meropenem/vaborbactam, are costly and are not integrated yet into routine antimicrobial susceptibility testing, are expensive, which restricts the available options for effectively treating CR-GNB infections [4], especially in resource-constrained settings.

All the CR-GNB isolates were multidrug-resistant (MDR) and had multiple antibiotic resistance (MAR) indexes ranging from 0.55 to 1.0. The MAR index is the ratio of antibiotics number an isolate is resistant to the total number of antibiotics used in susceptibility testing. The index is a good tool for health risk assessment. It helps to determine whether the isolates are from a region of high or low antibiotic use, with a MAR index greater than 0.2 indicating a ‘high-risk’ source of contamination [9]. This high MDR rates result in a substantial healthcare burden and are associated with increased mortality rates.

Patients with a history of antibiotic use were three times more likely to have CR-GNB isolate, consistent with other studies [26, 27] in China. Antibiotic exposure results in gut microbiota dysbiosis, involving a reduction in the diversity of gut microbiota, alterations in the abundance and gene expression, protein activity, and gut metabolome, compromised colonization resistance to invading harmful bacteria, and the emergence of antibiotic-resistant microbes [28]. This emphasizing the importance of implementing and upholding stringent antimicrobial stewardship practices to reduce overuse and reliance on specific antibiotic classes, especially carbapenems. We found patients using NG tubes to be six (6) times more likely to be diagnosed with GNB infection. Intubation disrupts the body’s natural protective barrier, allowing pathogens to invade, adhere and form biofilms in the inner surface of the tubes. The bacteria delivered to the gut by contaminated feeding tubes may lead to dysbiosis and poses significant health risks [29].

This study is subject to some limitations. First, the study utilized clinical laboratory-based CR-GNB testing using surveillance data, which may not accurately detect individuals who are carriers of these bacteria. Laboratory-based surveillance data may only capture cases where patients have symptomatic infections or during routine screening. This study may have missed colonized or asymptomatic carriers of CR-GNB, even though they can still contribute to the transmission and spread of CR within healthcare settings. Secondly, this was a single-centre study. As such, the findings may not accurately reflect the rate of CR in the region. However, the snapshot of cases reported is informative and can be very useful in estimating and comprehending the burden of carbapenem resistance in the hospital setting. Implementing comprehensive infection control measures, including active surveillance and screening of high-risk individuals, to effectively identify and manage carriers of CR-GNB can prevent transmission and outbreaks.

## Conclusion

Our study highlights high MDR- and CR-GNB infections in ICU, with prior antibiotic exposure and NGT use as risk factors, and diminishing clinical value of colistin and tigecycline. In this study setting and beyond, strict implementation of antimicrobial stewardship programs and adherence to infection prevention and control through monitoring, evaluation and feedback are warranted to curb CR-GNB infections, especially among risk groups.

## Data Availability

The datasets used and/or analysed during the current study are available from the corresponding author on reasonable request.
